# White Swelling of the Knee

**Published:** 1886-09-20

**Authors:** 


**White Swelling of the Knee.**—In a paper read before the Medical Society County of New York, Dr. Judson advocated the doctrine that it was essentially an inflammatory affection, and that an inflamed organ or tissue demanded arrest of function in the treatment, if the best results were obtained; that inflammatory conditions were relieved or removed by arrest of function, wherever it could be secured.

The essential feature of the treatment for diseases of joints should therefore, be fixation.

Prolonged fixation with disuse of a joint would not produce anchylosis, provided the joint itself was free from disease. Of course, it would be followed by stiffness, but that would yield by persistent passive movements, and was entirely different from anchylosis. The anchylosis which followed joint diseases, and was caused by the final products of inflammation, was best prevented by reducing or removing the inflammation, and to do this most effectually arrest of function was essential.

Fixation applied to a joint would, so far as the joint was free from disease, be powerless to add to the ultimate degree of anchylosis, and, so far as the joint was diseased, it would diminish the ultimate anchylosis by arresting inflammation and preventing an excess of its products.

On these premises thorough fixation was required in the treatment of articular ostitis. Dr. Judson thought it was impossible to establish the statement that motion was required to prevent inflammation.

In the treatment of joint disease, in the lower extremities particularly, another important function must be considered, namely, that of supporting weight and concussion. Protection of the articular surfaces from pressure and concussion was very important, and to accomplish this most certainly the best method was to convert the affected limb into a pendent member, putting it into very much the same condition, in this respect, as were the upper extremities.

When these indications had been thouroughly met, Dr. Judson believed that the patient had received the highest degree of assistance which surgery could afford.—*Epitome.*

